# Supplementary figures and images for: Location and expression kinetics of Tc24 in different life stages of Trypanosoma cruzi
Source: PLoS Negl Trop Dis. 2021 Sep 3;15(9):e0009689. doi: 10.1371/journal.pntd.0009689 (PMC8415617; doi:10.1371/journal.pntd.0009689)

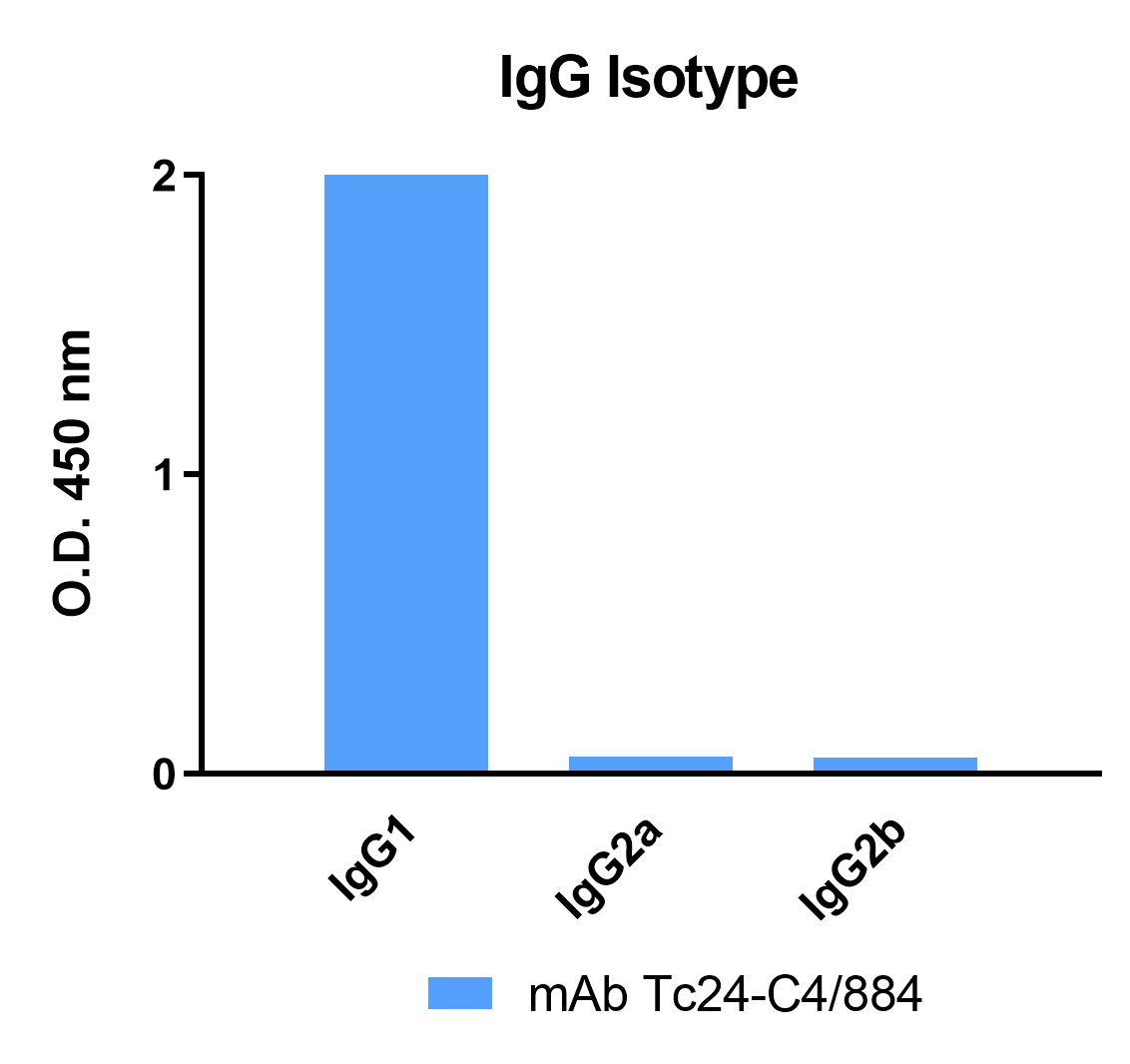

Supplement: S1 Fig — (TIF) [file pntd.0009689.s001.tif]

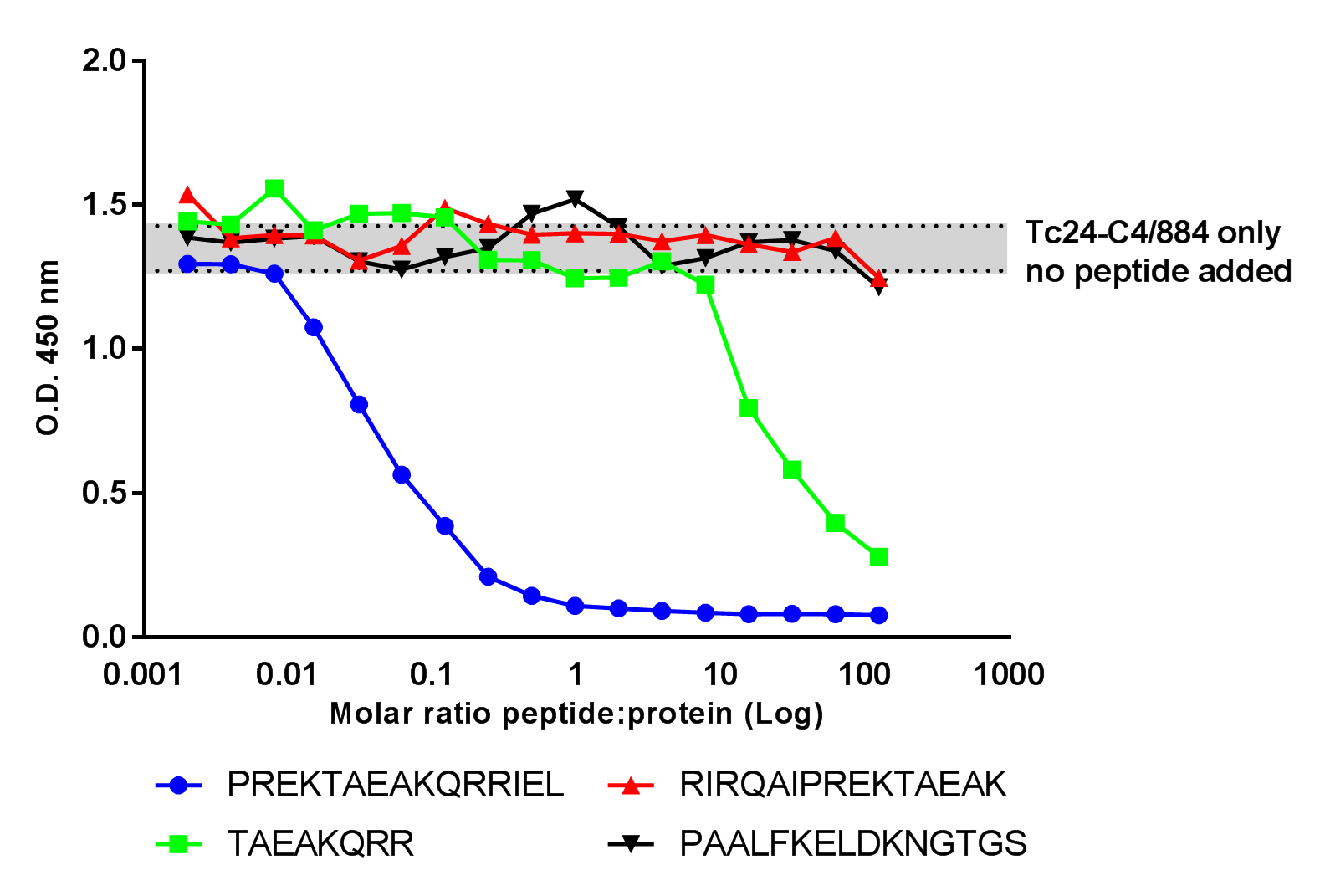

Supplement: S2 Fig — Four peptides were pre-incubated with Tc24-C4/884 mAb at different molar ratios followed by binding of Tc24-C4/884 on Tc24-C4 –coated ELISA plates. A decrease in O.D. 450 signal for Tc24-C4/884 pre-incubated with peptides PREKTAEAKQRRIEL and TAEAKQRR suggest that Tc24-C4/884 mAb binding sites were blocked and that sequence TAEAKQR(R) is the specific epitope of Tc24-C4/884. (TIF) [file pntd.0009689.s002.tif]

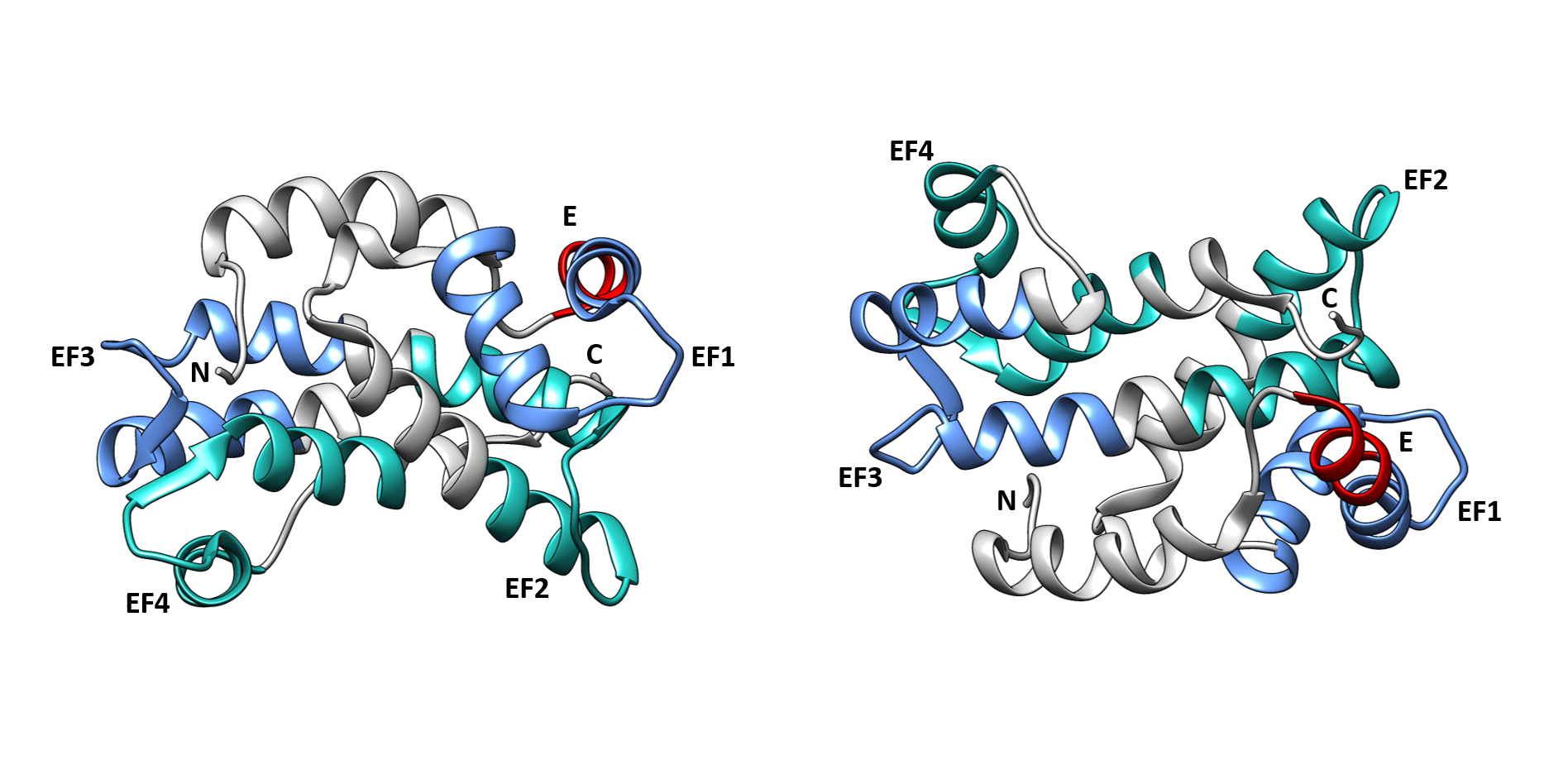

Supplement: S3 Fig — Epitope of Tc24-C4/884 is shown in red color. View from the membrane-binding interface (left image) and a horizontal 180° rotation (right image). Protein structure of Tc24 was obtained from Protein Data Bank in Europe (PDB code 3CS1) and rendered using USCF Chimera. E: epitope of mAb Tc24-C4/884 (in red). EF1: residues 49–77 (in blue). EF2: residues 98–126 (in cyan). EF3: residues 131–159 (in blue). EF4: residues 168–196 (in cyan).30 C: C-terminus. N: N-terminus. (TIF) [file pntd.0009689.s003.tif]

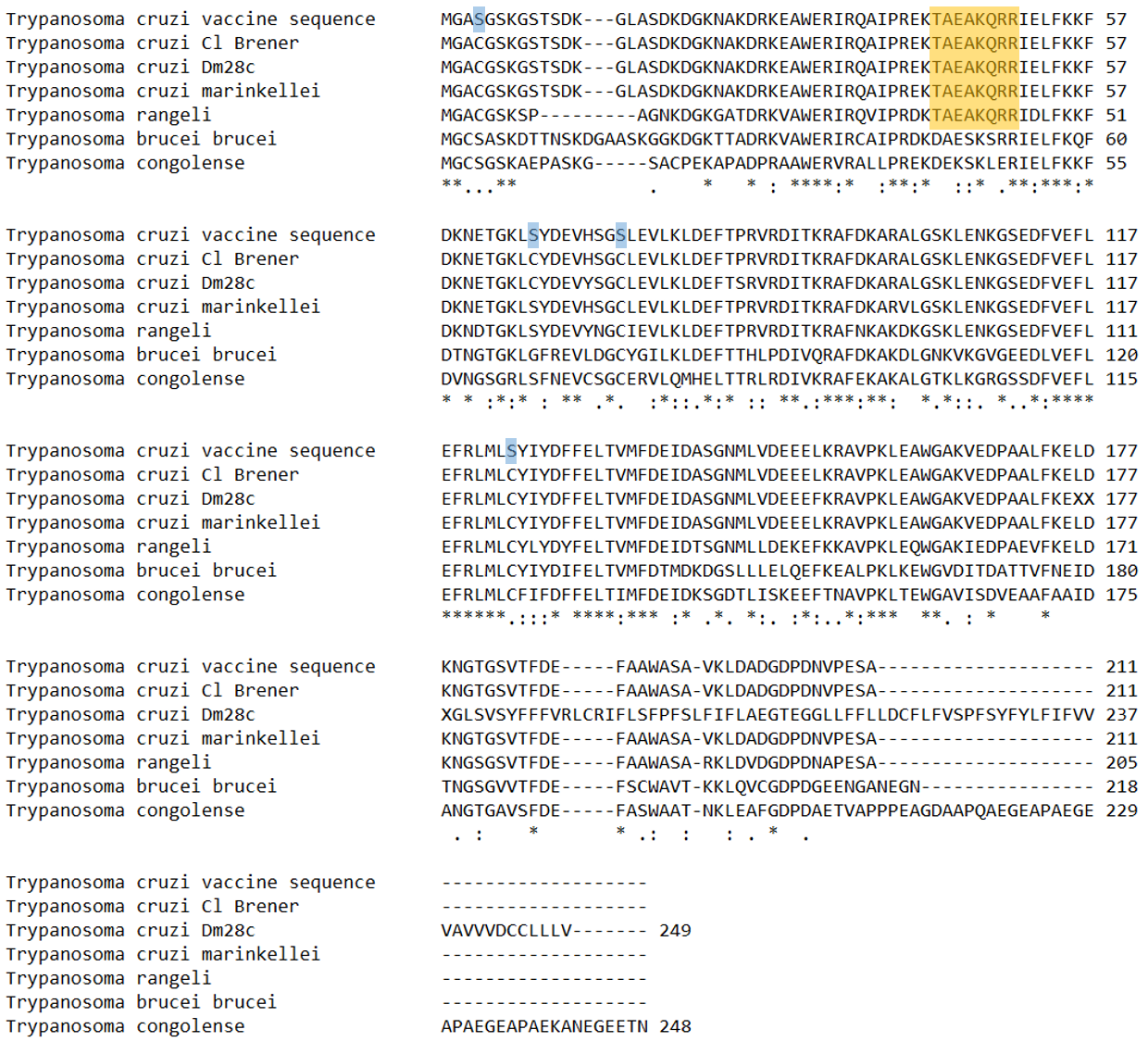

Supplement: S4 Fig — (TIF) [file pntd.0009689.s004.tif]

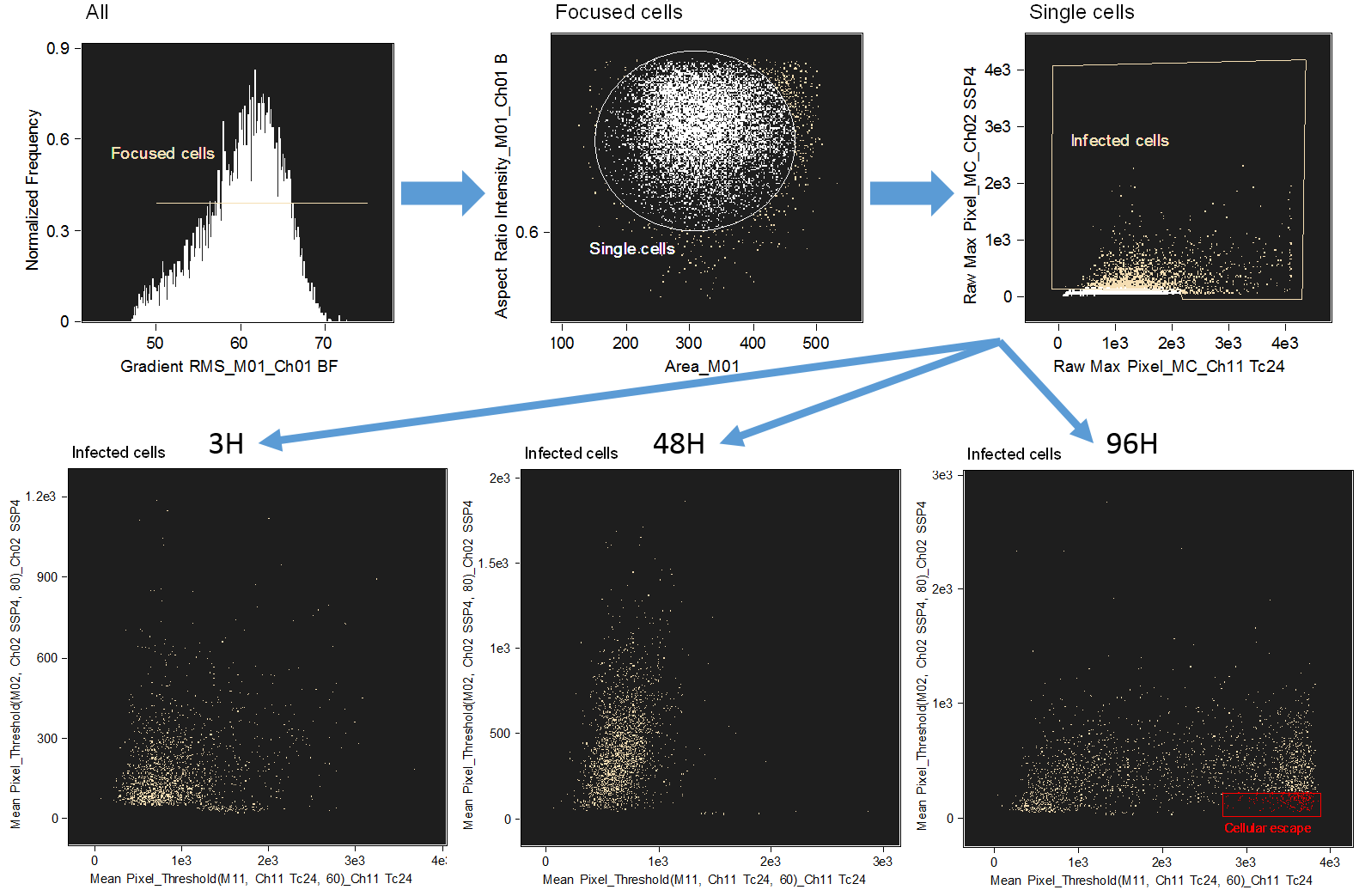

Supplement: S5 Fig — Events were first selected on focus using the gradient RMS feature followed by the selection of single cells only using the Area and Aspect Ratio features. The next gate involved the selection of cells which were infected by T. cruzi, which was achieved by gating around the cell population of an uninfected control sample. Finally, to remove background signal from “true” signal a Threshold mask was used for both Tc24 and SSP4 to select the area of interest to measure the MFI in. The features involving the Threshold mask for both Tc24 and SSP4 was plotted on a bivariate plot as shown in for 3 hrs, 48 hrs and 96 hrs. The MFI of the whole observed population was calculated and used in Fig 6A. By inspecting the images at 96 hrs, a manual gate was drawn which included events that expressed high MFI of Tc24 but no SSP4. (TIF) [file pntd.0009689.s005.tif]
