# Supplementary material for: Location and expression kinetics of Tc24 in different life stages of Trypanosoma cruzi
Source: PLoS Negl Trop Dis. 2021 Sep 3;15(9):e0009689. doi: 10.1371/journal.pntd.0009689 (PMC8415617; doi:10.1371/journal.pntd.0009689)
Supplement: Attachment — Submitted filename: PNTD-D-21-00386.docx [file pntd.0009689.s006.docx]

In this manuscript the authors studied a *T. cruzi* antigen describing its recognition by a monoclonal antibody (Mab) using different detection methods. Also, they mapped the potential B cell epitope. It’s a very interesting manuscript complementing the existing information on Tc24 *T. cruzi* antigen. Experiments are well conducted; the manuscript is well written with helpful information. Some additional information will be interesting: a competitive ELISA to determine specificity of the epitope, to use different methods of parasite fixation and a functional in vitro assay to determine the effect of the Mab during *T. cruzi* infection to susceptible cells.

Introduction:

- Mexico is not the country with the highest Chagas diseases prevalence in Latin America. Thus, it´s not clear the meaning of “specifically”.
- Amastigotes have an internal flagellum and trypomastigotes and external flagella.

Methods

- Refer to the official nomenclature and DTUs of the H1 strain.
- What percentage of trypomastigotes are obtained from the supernatant form infected VERO cells? Usually there are other parasite forms and they can be part of the antigen lysate.
- Please mention the ratio parasite/cells of infection for the culture assays

Results

- There is not surface detection of Tc24 in fixed trypomastigotes. Authors should test different fixation solution to rule it out the expression of Tc24 on the surface of trypomastigotes. Have the authors tested unfixed parasite?
- Figure 1a. There are no negative concentrations. It should be showed in a different manner.
- Figure 1b. Some weights should be indicated in the marker.
- It will be a complementary experiment to use a repetitive sequence of the TAEAKQR epitope in an ELISA competitive assay, with the most reactive overlapped peptides, to determine its specificity.
- Figure 3, 4 and 5. Please indicate magnification and use a bar for size reference.
- Figure 6. It is very difficult to read the text inside the figures, even using digital magnification, i.e. text inside imagining flow cytometry. It should have better quality.

Discussion

- “*Additionally, our microscopic analysis clearly shows that Tc24 is located along the whole flagellar membrane of trypomastigotes and is not concentrated in just the flagellar pocket, as earlier described*” This assumption is difficult to demonstrate with the images provide with the article. The Tc24 staining appears to be all around the cytoplasm of the parasite. A high magnification images to corroborate this phrase will be necessary.
- As discussed, TAEAKQR epitope is no part of the calcium-binding region, it will be useful to have this region marked in the supplementary Figure 2.

Finally, a functional assay will ideal and interesting. Using the anti-Tc24 Mab on trypomastigotes before and after infection of cultured cells would help to hypothesized mechanism of action if infection is inhibited.
