# Supplementary material for: Location and expression kinetics of Tc24 in different life stages of Trypanosoma cruzi
Source: PLoS Negl Trop Dis. 2021 Sep 3;15(9):e0009689. doi: 10.1371/journal.pntd.0009689 (PMC8415617; doi:10.1371/journal.pntd.0009689)
Supplement: Attachment — Submitted filename: Cover_letter_PLOSNTDs and responses to reviewers_V2.docx [file pntd.0009689.s007.docx]

**National School of Tropical Medicine**

Jeroen Pollet, Ph.D.

Assistant Professor

Baylor College of Medicine

1102 Bates St, Suite 550, Houston, TX 77030

Tel: +1 832-824-0545

E-mail jeroen.pollet@bcm.edu

June 28^th^, 2021

Dear Dr. Fujiwara,

Please find attached a revised version of our manuscript entitled: “*Location and expression kinetics of Tc24 in different life stages of Trypanosoma cruzi”,* intended as a contribution to PLOS NTDs.

We have responded to all of the reviewer`s comments and revised the manuscript accordingly. A list of the comments and the author`s responses can be found below this letter. We will also upload two versions of the revised manuscript: one with either highlights or tracked changes denoting where the text has been changed; the other a clean version (uploaded as the manuscript file).

The manuscript is original and no part of the manuscript has been published before, nor is any part of it under consideration for publication at another journal.

Thank you for your consideration,

Sincerely yours,

Jeroen Pollet, Ph.D.

Assistant Professor

Director of Tropical Medicine Innovations

Department of Pediatrics, Section of Tropical Medicine

National School of Tropical Medicine

Baylor College of Medicine

Response to Reviewers

**Location and expression kinetics of Tc24 in different life stages of Trypanosoma cruzi**

Leroy Versteeg, Rakesh Adhikari, Cristina Poveda, Maria Jose Villar-Mondragon, Kathryn M Jones, Peter J Hotez, Maria Elena Bottazzi, Edwin Tijhaar, Jeroen Pollet

The comments of the reviewers are listed below in a green font. The response of the authors to each question or remark is in a black font. The lines referred in the responses are from the clean document excluding the tracked changes.

**Reviewer #1**

**Methods**

*The methods are adequate and sufficient to complete the objectives of the study. Some suggestions from the reviewer are found in the attached review*

From attached document:

- *Refer to the official nomenclature and DTUs of the H1 strain.*

We have added the strain nomenclature and the DTU information to the manuscript.

- *What percentage of trypomastigotes are obtained from the supernatant form infected VERO cells? Usually there are other parasite forms and they can be part of the antigen lysate. Thank you for your question.*

We typically see no amastigotes in our parasite sample used for *T. cruzi* lysate. We harvest the trypomastigotes after their first round of cellular escape, which is around 5-6 days after infecting the cell culture with *T. cruzi*. We do observe an increase in extracellular amastigotes after 6 days.

- *Please mention the ratio parasite/cells of infection for the culture* *assays*

We have used a multipicity of infection (MOI) of 1 to 3 in these experiments. We have added this information into the manuscript.

*Finally, a functional assay will ideal and interesting. Using the anti-Tc24 Mab on trypomastigotes before and after infection of cultured cells would help to hypothesized mechanism of action if infection is inhibited.*

Thank you for the suggested experiment to improve our manuscript. We agree that it would be very interesting to further elucidate the mechanism of action of the humoral antibody response against Tc24 in T. cruzi infection. However, we believe that the effect of mAb Tc24-C4/884 in T. cruzi infection cannot be measured in a simple in vitro assay. We have shown that *T.cruzi* is not exposed on the surface of trypomastigotes, therefore the mAb will not be able to bind and trigger opsonization of the parasite. Based on our findings, the mAb can theoretically only interact with the soluble Tc24 or the Tc24 in EVs. To measure the effect of the mAb in these mechanisms, we need to have the interplay of different cells and tissues present in the experiment, which can only be done through an in vivo infection study. While we are planning to look further into this question, we think that this experiment is out of the scope of the current manuscript.

**Results**

*The results are well presented, some changes are suggested in the revision text.*

From attached document:

- *There is not surface detection of Tc24 in fixed trypomastigotes. Authors should test different fixation solution to rule it out the expression of Tc24 on the surface of trypomastigotes. Have the authors tested unfixed parasite?*

Thank you for your suggestion. For the fixation of the *T. cruzi* parasites and *T. cruzi* infected cells we have used a 4.2% formaldehyde buffer, which is commonly used to fix material prior to antibody staining. If the fixation buffer would negatively impact the binding of mAb Tc24-C4/884 to Tc24, then this would affect possible surface exposed as well as intracellular Tc24. However, we do observe good staining of Tc24 by Tc24-C4/884 in the formaldehyde fixed trypomastigotes after permeabilization with saponin, but not without permeabilization. Therefore, changing the fixation buffer would not change the observations made in this experiment, that Tc24 is expressed intracellular and not on the surface of trypomastigotes. Furthermore, due to biosafety restrictions, we cannot analyze live *T. cruzi* parasites or *T. cruzi* infected cells under the confocal microscope.

- *Figure 1a. There are no negative concentrations. It should be showed in a different manner.*

The concentrations of the mAb Tc24-C4/884 in this figure are shown in Log10 units. This was done because we tested over a wide range (20 ug/mL to 0.00000238419 ug/mL). In Log10, any value under 1 ug/mL will become negative. We changed the scale from ug/mL to ng/mL, so that all the values remained about 1 and no negative values are shown.

- *Figure 1b. Some weights should be indicated in the marker.*

Thank you for your suggestion, we have added the molecular weights of the bands of the marker in the Figure.

- *It will be a complementary experiment to use a repetitive sequence of the TAEAKQR epitope in an ELISA competitive assay, with the most reactive overlapped peptides, to determine its specificity.* We appreciate your suggestion and have conducted a competitive ELISA to further confirm the specificity of mAb Tc24-C4/884 to epitope TAEAKQR(R). The ELISA results are shown in supplementary Figure S2, and the methods are described under “Competitive ELISA”. The data are discussed in lines 323-328.

The epitope microarray technology is rapidly becoming a standard technique to discover the linear epitope of monoclonal antibodies (Heiss et all., 2020). The array results show that there is no sequence starting before TAEAKQRR to which Tc24-C4/884 mAb binds to, as well as no sequence after TAEAKQR(R) (Figure 2B). In combination with the new ELISA data, we feel confident to conclude that the mAb binds specifically to the TAEAKQR(R) epitope of Tc24-C4/884.

- *Figure 3, 4 and 5. Please indicate magnification and use a bar for size reference.*

Thank you for your suggestion for improvements. We have added the scale bars in the respective figures. The instrument used in this study is a Leica laser point scanning confocal system, where light is collected by a detector and not a camera. As a consequence, the image size is variable and magnification is typically expressed in terms of pixel size and not as a total image magnification like one would for a camera based system with fixed pixel size. We have added the pixel size in the legend of each figure.

- *Figure 6. It is very difficult to read the text inside the figures, even using digital magnification, i.e. text inside imagining flow cytometry. It should have better quality.*

We have improved the image quality of the figures so that the text is easier to read.

**Conclusions**

*The conclusions are adequate. Although, two specific changes to the discussion are suggested.*

From attached document:

*“Additionally, our microscopic analysis clearly shows that Tc24 is located along the whole flagellar membrane of trypomastigotes and is not concentrated in just the flagellar pocket, as earlier described” This assumption is difficult to demonstrate with the images provide with the article. The Tc24 staining appears to be all around the cytoplasm of the parasite. A high magnification images to corroborate this phrase will be necessary.*

Thank you for your suggestion. Previous publications described that Tc24 is only located in the flagellar pocket which suggests that mAb Tc24-C4/884 would only bind in that specific region which is located at the posterior of the trypomastigote. However, our observations showed that Tc24 was located along the whole parasite and therefore is not only located in the flagellar pocket. Our findings support the findings of other researchers which described that Tc24 is located on the flagellar membrane, and we believe our conclusions are therefore correct. Furthermore, it has been described that Tc24 indeed can be present in the cytoplasm. Tc24 is a calcium switch protein which binds to the inner leaflet of the flagellar in the presence of calcium. When calcium is not present, or artificially removed by washing parasites with EDTA, Tc24 will not associate with the flagellar membrane and can be found in the cytoplasm. In the “normal” state of the parasite there is calcium present and most of the Tc24 will be associated to the flagellar membrane. (Godsel, L. M., and D. M. Engman. The EMBO Journal 18, no. 8 (April 15, 1999): 2057–65).

*As discussed, TAEAKQR epitope is no part of the calcium-binding region, it will be useful to have this region marked in the supplementary Figure 2.*

The epitope TAEAKQRR is marked in supplementary Figure 2 with a red color and the letter “E” of epitope.

**Editorial and Data Presentation Modifications**

(No Response)

**Summary and General Comments**

*This is a well conducted and presented study. It is suggested in the possible two experiments to demonstrate the specificity of the described epitope and a functional test with the monoclonal antibody in infection assays with cell cultures*.

Thank you, we appreciate your suggestions.

From attached document, general comment:

*In this manuscript the authors studied a T. cruzi antigen describing its recognition by a monoclonal antibody (Mab) using different detection methods. Also, they mapped the potential B cell epitope. It’s a very interesting manuscript complementing the existing information on Tc24 T. cruzi antigen. Experiments are well conducted; the manuscript is well written with helpful information. Some additional information will be interesting:*

*a competitive ELISA to determine specificity of the epitope.* we have addressed this suggestion in the “Results” section on page 3, *to use different methods of parasite fixation* We have address this suggestion in the “Results” section on page 3,

*and a functional in vitro assay to determine the effect of the Mab during T. cruzi infection to susceptible cells.* We have addressed this suggestion in the “Methods” section on page 2.

*Finally, a functional assay will ideal and interesting. Using the anti-Tc24 Mab on trypomastigotes before and after infection of cultured cells would help to hypothesized mechanism of action if infection is inhibited.* We have addressed this suggestion in the “Methods” section on page 2

From attached document, introduction related:

- *Mexico is not the country with the highest Chagas diseases prevalence in Latin America. Thus, it´s not clear the meaning of “specifically”.*

Thank you for catching this incorrect statement, we have removed the sentence.

- *Amastigotes have an internal flagellum and trypomastigotes and external flagella.*

We have improved our definitions of the flagella in the introduction.

**Reviewer #2**

**Methods**

*The objectives of the study are clearly articulated with a clear testable hypothesis. The study design and the population are appropriate clearly described  to address the stated objectives. The samples size is sufficient and the statistical analysis used support conclusions. There are no concerns about ethical or regulatory requirements being met.*

We are pleased to hear that you are satisfied with the methods used in our manuscript.

**Results**

*The results are clearly presented and match the analysis plan. The figures have a good resolution and allow to identify the results. Although figure 3 allows to view the results, its resolution can be increased.*

Thank you for your suggestion. We have increased the resolution of Figure 3 to improve the quality of the results.

**Conclusions**

*The conclusions are supported by the presented data. The limitations of the analysis can be better described and discussed in more detail. The authors discuss how the data can be helpful to advance our understanding of the topic under study.*

We appreciate your comments. We have added some limitations of the analysis in the discussion of the manuscript (line 501).

**Editorial and Data Presentation Modifications**

*The authors present many studies of Tc24 as a vaccine candidate in the introduction of the manuscript. In most of the discussion, the authors emphasize that the protein is inside T. cruzi, requiring permeabilization. This is really important, the main conclusion of the study. However, they do not discuss much about how it can affect immunization. The discussion regarding the immune response should be broader, addressing more work. The authors' results are good and may help in other studies, but they need to discuss further the Tc24 relationship and immune response.*

We appreciate your supportive feedback. We have re-organized the discussion on Tc24 vaccine-induced protection and we added more discussion to strengthen our conclusions and hypothesis.

**Summary and General Comments**

(No Response)

**Reviewer #3**

**Methods**

*The main goal of the work is to better characterize the expression and location of a T. cruzi vaccine candidate antigen, Tc-24, by using a monoclonal antibody. To my knowledge, all the experiments were carefully designed, with all the appropriate controls and statistical analysis, and mainly adequate to the purpose of the investigation: level of protein expression and localization of the protein within the vertebrate life cycle forms of the parasite, trypomastigotes and amastigotes. One experiment to validate level of protein expression could be performed to support the data. There are no concerns about ethical and regulatory requirements.*

Thank you for your supportive comments.

**Results**

*As mentioned, the experiments were adequately designed, as well as clearly presented (text and figures). There is a result from figure 4 there is not clear though. They show in the figure “diffrente” stages of amastigotes in the same cell, it was not clear how they determined this. Some other comments about the results and an experiment that should be performed to support data from protein expression levels between the two parasite stage life cycles are mentioned in the “summary and general comments”.*

Thank you for the kind suggestions. We have addressed these in the “summary and general comments” section on page 6.

**Conclusions**

*The main conclusions are supported by the results. The authors also discuss how the data can be helpful to advance our knowledge. However, some points of the discussion should be reviewed or further addressed. This has been mentioned in the “summary and general comments”.*

We appreciate your input. We have addressed your suggestions in the “summary and general comments” section on page 6.

**Editorial and Data Presentation Modifications**

*Modifications or experimental suggestions are presented in the “summary and general comments”. No other minor modifications are necessary.*

Thank you.

**Summary and General Comments.**

*The main goal of the work was to better characterize the expression and location of a T. cruzi vaccine candidate antigen, Tc-24, by using a monoclonal antibody. All the experiments were carefully designed, with all the appropriate controls and statistical analysis, to my knowledge, to investigate the level of expression and localization of the protein within the vertebrate life cycle forms of the parasite, trypomastigotes and amastigotes. The main results of the manuscript are: the differential expression of Tc-24 between the two vertebrate life forms of the parasite and the fact that Tc-24 is not exposed to the outside of the flagellar membrane, which could question its use for a vaccine. However, some important points should be raised.*

Thank you for your supportive feedback.

*- It has long been shown that Tc-24 is a flagellar calcium binding protein (Engman et al., J. Biol. Chem., 264, 18627–18631, 1989), linked to the membrane via calcium induced miristoylation and palmitoylation (Godsel and Engman, EMBO J. Apr 15;18(8):2057-65, 1999). Although Engman and coworkers, in the manuscript from 1989, had shown that Tc24 is expressed in all life forms of T. cruzi with different levels, here they show with different experimental approaches that its expression is reduced, at least in some stages of the amastigote form of the parasite. Nonetheless, this should be expected, since it had been previously shown, as mentioned, that Tc-24 is a flagellar calcium binding protein, mainly expressed in the flagellar membrane. As it is well known, amastigote forms present just a flagellar remnant. if the authors intended to prove that amastigotes really show reduced expression of Tc-24, it would be important to quantify the level of expression by performing a western blot of protein extracts of the two purified forms, using the Tc-24mAb.*

Based on the reviewer`s suggestion, we have prepared lysate from isolated intracellular amastigotes and compared this on western blot with lysate from isolated trypomastigotes for the presence of Tc24. We added the results as Figure 5 in the manuscript and the methods are described in the methods section. A Coomassie Brilliant Blue stained gel was added to the figure to show that the same amount of lysate was loaded on the gels. The results confirmed the observations made in the microscopic images. While there was a very limited amount of Tc24 detected in the amastigote lysate, this band on western blot was very faint compared to the band of the trypomastigote lysate.

*-In figure 4 the authors recon there are different amastigote stages on the same cell. However how they defined it or if this was only determined by Tc-24mAb labeling is not clear. The authors must make this point clearer in the text.*

Thank you for your suggestion to improve our manuscript. Aside of our observation that there is a difference in stage III and stage IV in the amount of expression of Tc24, a difference in the organization of kinetoplasmid DNA (kDNA) can be observed in the Cytrak Orange channel. It is described that in amastigotes the kDNA is tightly packed and looks like a disk-shape, while trypomastigotes have a less dense kDNA organization and this disk-shape is not observed (Cavalcanti et all. BMC Microbiology 9 (June 4, 2009): 120). In *T. cruzi* parasite stage III, we observed a less dense-organized kinetoplast, which suggests that this parasite is not fully transitioned to the amastigote stage, yet. Therefore, we believe that there are different amastigote stages within the same cell. We have added this explanation to the manuscript for clarification.

-*The results from figures 4 and 6 are incongruent. On figure 4 they show that parasites that had just completed transitioned to amastigote still express Tc-24, its expression drastically decreases on fully differentiated dividing amastigote. On figure 6 the lower expression levels of Tc-24 is observed for intracellular parasites 24 hours post infection, which should correspond to trypomastigotes that had just transitioned to fully amastigotes. 48 and 96 hours later (when amastigotes are dividing), Tc-24 expression levels increases again. The same is true for the 96 hours post infection, even though at this time point there are also parasites that have transitioned back to trypomastigotes. The authors must revise this data or at least explain it better in the text and discus it further in the discussion section. Additionally, as mentioned before, if the authors intended to prove that amastigotes really show reduced expression of Tc-24, it would be important to quantify the level of expression by performing a western blot of protein extracts of the two purified forms, using the Tc-24mAb.*

Thank you for your constructive feedback. While both Figure 4 and Figure 7 (was Figure 6) show a decrease in Tc24 expression after the trypomastigotes transform into amastigotes, it is a correct observation that in figure 4 it looks like there is no Tc24 expression in the replicating amastigotes (stage IV). The reason for this difference is that the imaging technologies used are different, and they capture light emitted by fluorophores differently. Whereas confocal microscopy captures emitted light from a very thin slice of the sample (referred as the z-axis), with imaging flow cytometry (IFC) the emitted light from the complete sample is acquired. Therefore, more light is captured in IFC and more Tc24 signal is detected. If we would acquire the images in figure 4 using the confocal microscope at different slices, the images could be combined and the Tc24 signal would accumulate, resulting in the dim Tc24 signal in amastigotes as observed with IFC. In fact, figure 7 was prepared by merging different imaging slices, resulting in a visible dim signal in the amastigote population in picture A3. Unfortunately, at this point we are not able to retrieve the exact location of the cells and parasites on the microscopic slides for figure 4, so we can’t re-acquire this image. We will address the difference in imaging techniques and the difference in Tc24 signal between Figure 4 and 7 in the manuscript for clarification.

*-In the manuscript from Godsel and Engman (1989) they had also shown that Tc24 is a member of the calcium–acyl switch protein family, therefore binding to the inner leaflet of the plasma membrane, which was corroborated later (Maric et al. J Biol Chem. 286(38): 33109–33117, 2011). Therefore, should be no surprise that labeling with Tc-24 mAb would only recognize Tc 24 upon fixation and permeabilization. Also in the discussion they mentioned that it has been previously suggested that the protein was located in the outside of the membrane, but this was based only in the fact that infection raises antibodies to this protein. However, as far as I am concerned, the first mention of this protein in the literature was as a member of the excretory/secretory product, so it is possible that this protein may be secreted when it is not bound to the flagellar membrane. In fact the authors even mention this in the discussion.*

Thank you for your remarks. We agree with your train of thought on the location of Tc24. After investigating our findings we found that in the Chagas disease vaccine research field it is often stated that Tc24 is located in the flagellar pocket of the parasite, and that antibodies against Tc24 could bind to parasites. When one would search the parasitology literature for the location of flagellar Calcium-binding proteins (FCaBPs) however, there is much known on the location of Tc24 and other calcium sensing proteins. We believe that the usage of two names for the same protein caused the vaccine field to overlook some publications, and we hope by publishing this manuscript we merge the fields together.

*-Since the authors raise the point that this characterization is important for addressing the actual viability and role of this protein as a vaccine candidate, it would be important also to perform the labeling of purified proteins from parasite culture supernatant to verify whether it is really found as a secreted protein.*

Thank you for your suggestion. While we agree that it is very interesting to test our hypotheses to see whether T. cruzi trypomastigotes secrete soluble Tc24 or in extracellular vesicles, we think this is out of the scope of this manuscript. In this manuscript we report novel monoclonal antibodies that recognize a linear epitope of Tc24, which we then used to detect the location and presence of Tc24 in trypomastigotes and T. cruzi infected cells using different imaging techniques. We found that Tc24 is not located on the surface on trypomastigotes, and that the expression of Tc24 is different between trypomastigotes and amastigotes. We then discuss our observations in line with the hypothesized mechanisms of Tc24 vaccine-induced protection. This manuscript was not written with the goal to test our newly made hypotheses by conducting more experiments. However, we agree that for a future manuscript we could try to test our new hypotheses and to further improve our understanding of the mechanisms of Tc24 vaccine induced protection. Fellow researchers have already been successful in obtaining EVs from trypomastigotes in culture (Lovo-Martins et all., Frontiers in Immunology 9 (April 27, 2018)) and the methodology to detect Tc24 in EVs or soluble Tc24 has already been describes as well (Bayer-Santos et all., Journal of Proteome Research 12, no. 2 (February 1, 2013): 883–97).
